# Kidney volume measurement methods for clinical studies on autosomal dominant polycystic kidney disease

**DOI:** 10.1371/journal.pone.0178488

**Published:** 2017-05-30

**Authors:** Kanishka Sharma, Anna Caroli, Le Van Quach, Katja Petzold, Michela Bozzetto, Andreas L. Serra, Giuseppe Remuzzi, Andrea Remuzzi

**Affiliations:** 1 Bioengineering Department, IRCCS Istituto di Ricerche Farmacologiche Mario Negri, Bergamo, Italy; 2 Zurich Center for Integrative Human Physiology, University of Zurich, Zurich, Switzerland; 3 Epidemiology, Biostatistics and Prevention Institute, University of Zurich, Zurich, Switzerland; 4 Unit of Nephrology and Dialysis, ASST Papa Giovanni XXIII, Bergamo, Italy; 5 Department of Biomedical and Clinical Sciences, University of Milan, Milan, Italy; 6 Department of Management, Information and Production Engineering, University of Bergamo, Bergamo, Italy; UCL Institute of Child Health, UNITED KINGDOM

## Abstract

**Background:**

In autosomal dominant polycystic kidney disease (ADPKD), total kidney volume (TKV) is regarded as an important biomarker of disease progression and different methods are available to assess kidney volume. The purpose of this study was to identify the most efficient kidney volume computation method to be used in clinical studies evaluating the effectiveness of treatments on ADPKD progression.

**Methods and findings:**

We measured single kidney volume (SKV) on two series of MR and CT images from clinical studies on ADPKD (experimental dataset) by two independent operators (expert and beginner), twice, using all of the available methods: polyline manual tracing (reference method), free-hand manual tracing, semi-automatic tracing, Stereology, Mid-slice and Ellipsoid method. Additionally, the expert operator also measured the kidney length. We compared different methods for reproducibility, accuracy, precision, and time required. In addition, we performed a validation study to evaluate the sensitivity of these methods to detect the between-treatment group difference in TKV change over one year, using MR images from a previous clinical study. Reproducibility was higher on CT than MR for all methods, being highest for manual and semiautomatic contouring methods (planimetry). On MR, planimetry showed highest accuracy and precision, while on CT accuracy and precision of both planimetry and Stereology methods were comparable. Mid-slice and Ellipsoid method, as well as kidney length were fast but provided only a rough estimate of kidney volume. The results of the validation study indicated that planimetry and Stereology allow using an importantly lower number of patients to detect changes in kidney volume induced by drug treatment as compared to other methods.

**Conclusions:**

Planimetry should be preferred over fast and simplified methods for accurately monitoring ADPKD progression and assessing drug treatment effects. Expert operators, especially on MR images, are required for performing reliable estimation of kidney volume. The use of efficient TKV quantification methods considerably reduces the number of patients to enrol in clinical investigations, making them more feasible and significant.

## 1. Introduction

Autosomal dominant polycystic kidney disease (ADPKD) is characterized by the development of fluid-filled cysts leading to progressive kidney volume (KV) enlargement despite apparently normal renal function up to 40–50 years of age [1]. The increase in KV, which is followed by renal function decline only at a later stage [2–4], is widely accepted as the dominant feature of ADPKD progression and provides a metric of disease progression [5–7]. Besides disease progression, the estimation of total kidney volume (TKV) has been extensively used in the past to investigate the effect of pharmacological treatments in ADPKD patients [8–19]. Clinical studies have highlighted that drug treatment in these patients could limit kidney enlargement earlier and to a greater extent than slowing glomerular filtration rate (GFR) decline [7].

Recently, TKV has been increasingly gaining acceptance for two important reasons. First, as biomarker for classification of disease severity, at individual patient level, for enrollment in clinical studies [20–22]. Second, TKV is used as end-point of therapeutic efficacy in these ADPKD clinical trials [7, 23]. The current and potential use of TKV has particularly brought to attention the variable choice of imaging modalities and methods employed for calculating TKV in several studies reported on ADPKD [5, 18, 24–28].

TKV can be measured on either magnetic resonance (MR) or computed tomography (CT) [5, 24, 25] with methods that differ in complexity, time required, accuracy, and precision. Until now, the most commonly used methods include whole kidney contouring (hereafter named as planimetry)[18] and Stereology (grid point counting over the kidney) [26]. Since these methods are time consuming, simpler and faster methods using a mid-slice approach or an ellipsoid equation have been proposed to shorten the time required to estimate TKV [27, 28]. In a recent study, Spithoven and coworkers suggested that such methods are equivalent to manual planimetry for classification of disease severity in ADPKD patients [29]. As pointed out by these studies [28, 29], to identify patients with a high likelihood of rapid disease progression for enrolment in clinical studies, extremely precise methods for TKV estimation are not required, and these simplified methods may be used. However, to use TKV as end-point to investigate the effects of drug treatments, KV measurements must be precise and accurate, to effectively detect small changes that develop over time intervals as short as 6 months or 1 year. Additionally, the use of precise measurements is necessary to limit the number of patients to enrol, thus making clinical studies more feasible and significant. In previous longitudinal studies the mean rate of TKV growth per year in patients receiving standard of care ranged from 5.3% to 5.7% [2, 7, 8, 30]. TKV growth can decrease to less than 3% per year in patients under treatment [8, 12, 17]. Thus, it is imperative to know the true precision and accuracy of the method to adopt for TKV computation for detection of these small TKV changes. In addition, it is necessary to consider the reproducibility and time required for measuring TKV.

Previous studies assessed the validity of single or a couple of TKV estimation methods in comparison with either manual planimetry or Stereology [26–29, 31, 32]. However, to date no study formally compared precision, accuracy and reproducibility, as well as the amount of time required by different methods used for TKV measurement in ADPKD. A comprehensive comparison of these methods is crucial to define the adequacy of TKV quantification strategies in clinical investigations that aim to evaluate the effect of drug treatments. The aim of our investigation was then to systematically compare the different methods available for quantification of TKV on both MR and CT images acquired within clinical studies on ADPKD. We also investigated the influence of expertise required by each method. We finally performed a validation study to evaluate the sensitivity of each method to detect the difference in TKV change over 1-year period between two treatment groups, using MR images from a previous clinical study.

## 2. Methods and materials

### 2.1 Study design

In this study, we first systematically compared different methods available for TKV quantification in terms of reproducibility, accuracy, precision, and time required, on a series of MR and CT acquisitions (hereafter named as experimental dataset) obtained within two clinical studies on ADPKD. Two independent operators with different level of experience computed the volume of single kidneys (SKV) on all MR and CT images, using the different available methods. The expert operator (KS for both MR and CT) routinely performed KV computations for ADPKD clinical trials for two years, having experience with all different techniques, while the beginner operators (KP for MR and LVQ for CT) started to perform KV computation for the purposes of the current study after specific training on kidney anatomy and computational methods. SKV values, instead of TKV ones, were used to estimate the true accuracy and precision of volume estimations to avoid the averaging effect of the two volume measurements on potential error occurring in TKV calculation. Each operator computed SKV twice, at least two weeks apart, to eliminate potential memory of previous measurements. We then performed a validation study on MR images from another clinical study (ALADIN [12]) to assess the sensitivity of individual TKV quantification methods to detect the difference in TKV change over 1-year period between two treatment groups.

### 2.2 MR and CT image sources for the experimental dataset

All MR and CT images included in the experimental dataset were obtained from previously performed and on-going clinical trials in patients with typical ADPKD (class 1). The ethical approval was obtained for all studies by local ethics Committees (ALADIN study: “Ospedali Riuniti di Bergamo” (del. n. 214, 20/02/2006), “Unità Sanitaria Locale Le/1” (del. n. 1236, 5/5/2006), “Fondazione Ospedale Maggiore Policlinico Mangiagalli e Regina Elena” (prot. n. 117, 19/01/2007), “Università degli studi di Napoli Federico II” (prot. n. 22868, 3/7/2007), “Provincia di Treviso” (del. n. 1317, 28/11/2007); ALADIN2 study: “ASL della provincia di Bergamo” (approved on 16/03/2011), “Azienda sanitaria locale di Agrigento (del. n. 4178, 14/06/2012), “Azienda sanitaria locale di Lecce” (del. n. 1836, 7/10/2011), “Fondazione IRCCS Ca' Granda Ospedale Maggiore Policlinico di Milano” (prot. n. 2336, 16/09/2011), “Università degli studi di Napoli Federico II (prot. n. 0012523, 12/08/2011), “Provincia di Treviso” (del. n. 375, 13/04/2012); SIRENA2 study: “Ospedali Riuniti di Bergamo” (del. n. 1884, 24/12/2007); EUROCYST study: “Provincia di Bergamo” (del. n. 132/2014, 30/01/2014)). The patients provided written consent for their medical records to be used in research studies at clinical study enrolment. Data were accessed anonymously.

MR images were baseline examinations of 15 ADPKD patients enrolled in the EuroCYST study [33], a multi-centre longitudinal observational study on ADPKD progression in patients with estimated GFR≥30 ml/min/1.73m^2^ (clinicaltrials.gov identifier NCT02187432). MRIs were acquired according to the EuroCYST MRI acquisition protocol [33], including standard localizer, T2 single shot fast/turbo spin echo (coronal acquisition, 4 mm slice thickness, 0 mm spacing, FOV = 30–35 to avoid wrap-around, 256 x 256 matrix, TE ≈ 70–190 ms based on the vendor and max TR), FISP or FIESTA 3D spoiled gradient echo (coronal acquisition, 4 mm slice thickness, 0 mm spacing, FOV = 30–35, 256 x 256 matrix, TE ≈ 2 ms, TR ≈ 7 ms, flip-angle = 40–50°), and T1-3D spoiled gradient echo (coronal acquisition, slice thickness of 4mm, spacing 0mm, FOV = 30–35, 256 x 256 matrix, TE ≈ 2 ms, TR ≈ 4 ms, flip-angle≤15°). Once acquired, MR images were transferred to DICOM 16-bit format from the clinical scanner on digital media, and 3D-T1 MRI sequences were used for KV computation. 3D-T1 MR images included in this study (n = 15) were taken from six different centres of the EuroCYST study and were selected to uniformly represent a large range of single KV (from 707 to 6605 ml) and different image quality.

The CT images were acquisitions from ADPKD patients with estimated GFR≤40 ml/min/1.73m^2^ enrolled in either ALADIN 2 (clinicaltrials.gov identifier NCT01377246) or SIRENA-II [19] (clinicaltrials.gov identifier NCT01223755) clinical trials. These CT images were acquired in a single breath-hold scan (120 kV; 150 to 500 mAs; matrix 512x512; 2.5 mm collimation; 0.984 slice pitch; 2.5 mm increment). Each CT acquisition was initiated 80 seconds after the infusion of 100 ml non-ionic iodinated contrast agent (Iomeron 350; Bracco, Italy) at a rate of 2 ml/s, followed by 20 ml saline solution at the same infusion rate. Once acquired, CT images were transferred in DICOM 16-bit format from the clinical scanner on digital media, and resampled to 5 mm slice thickness for KV computation. The CT acquisitions used in this study (n = 5 from SIRENA-II and n = 10 from ALADIN 2 clinical studies) were taken from different centres and were selected to uniformly represent a large single KV range (from 598 to 6002 ml) and different image quality.

The main socio-demographic and clinical features of ADPKD patients included in the experimental data set are reported in Table 1.

**Table 1:** Demographics and clinical characteristics of ADPKD patients included in the experimental and validation datasets, from past and on-going clinical trials.

	Experimental dataset		Validation dataset
	MR	CT	MR
n	15	15	75
Clinical study	EuroCYST	SIRENA-II (n = 5) ALADIN 2 (n = 10)	ALADIN
Age (years)	49 [38–62]	51 [35–67]	37 [20–63]
Gender (females)	7 (47%)	4 (27%)	39 (52%)
GFR (mL/min/1.73m^2^)	62 [31–114]^#^	22 [10–35]	84 [32–137]^§^
Left KV (ml)	1,474 [365–3,061]	1,558 [335–3,184]	971 [186–2,634]
Right KV (ml)	1,366 [308–3,544]	1,596 [263–3,256]	877 [169–3,317]
Total KV (ml)	2,840 [707–6,605]	3,154 [598–6,2]	1,855 [404–5,577]

Demographic and renal function parameters.

Abbreviations: GFR, glomerular filtration rate; KV, kidney volume.

^#^missing data for n = 3 patients

^§^missing data for n = 2 patients

GFR was estimated by MDRD equation (experimental dataset), or measured by Iohexol plasma clearance (validation dataset)

Values are expressed as mean [range] or number (%)

### 2.3 Kidney volume computation methods

SKV was computed on a series of 30 kidneys for each imaging modality (experimental dataset) using all of the methods described hereafter. Additionally, the expert operator also measured the kidney length. At the main renal blood vessels and hilum, the kidney outline was defined making a straight line between the parenchyma lips. Fat and vessels lying inside the kidney were included in the outline, while fat surrounding the kidney was excluded. Special attention was paid to regions where kidneys and liver were adjacent.

#### 2.3.1 Polyline manual tracing

To obtain an accurate measure of SKV, the kidney contour was manually segmented using the polygon tool of ImageJ software [34] (NIH, Bethesda, MD). Each kidney was outlined by manually drawing a polyline composed of numerous points on all contiguous slices. SKV was computed as the sum of the surface area of all the kidney outlines, multiplied by the slice thickness. This planimetry method is later referred to as “ImageJ polyline” method. The theoretical accuracy of planimetry in quantifying the volume of an object with ellipsoidal shape, based on the area of serial sections, depends on section thickness and orientation with regard to the object size. To estimate the volume quantification error caused by sectioning, we considered three ellipsoids of different sizes, and applied planimetry, using randomly positioned and uniformly distributed serial sections with thickness and orientation typical of MR and CT imaging. As shown in the Supporting Information (S1 File), the volume quantification error of theoretical planimetry, in comparison with analytical volume, is less than 0.26% and 0.10% for MR and CT sectioning, respectively. On the basis of these results we deduced that manual segmentation of kidney outlines on serial sections by polylines could represent the reference method for KV computation.

#### 2.3.2 Free-hand manual tracing

To evaluate a faster method of kidney segmentation, which does not require the placement of consecutive points on the kidney border, each kidney was traced by free-hand drawing of its outline on all contiguous slices using Osirix imaging software [35], an image processing software that runs on Mac OS X only. Similarly to polyline, SKV was computed as the sum of the surface area of all the kidney outlines, multiplied by slice thickness. This planimetry method was later referred to as “Osirix free-hand”.

#### 2.3.3 Semi-automatic manual tracing

This semi-automatic outline tool was designed to further reduce KV quantification time. We made use of a plugin in ImageJ software based on the livewire segmentation (ivussnakes.sourceforge.net)[36] and customized it for outlining the polycystic kidneys on all contiguous slices. Starting from a manually selected seed point, the Livewire tool automatically identifies the kidney boundary while the operator moves the mouse over the region of interest. Up to the point that the tool recognizes the correct boundary segment, the operator places a new seed point to confirm the selection, and this process is repeated until the kidney has been completely segmented. Then SKV was computed as sum of the areas of the kidney outlines, multiplied by the slice thickness. This planimetry method was later referred to as “Livewire tool”.

#### 2.3.4 Stereology

Each kidney was segmented using a stereology technique, by counting the number of intersections of a randomly positioned grid over the organ [26]. Stereology was performed using the ImageJ Grid plugin (rsb.info.nih.gov/ij/plugins/grid.html), the grid comprising crosses placed on the 3D stack with 16 x 16 mm grid spacing, 16 mm slice thickness for MR images and 15 x 15 mm spacing, 15 mm slice thickness for CT images. A random offset was used for grid position. Grid spacing was set after testing several values, in order to reduce time required while maintaining accuracy. SKV was computed as point count, multiplied by grid square area and by slice thickness.

**Fig 1:**
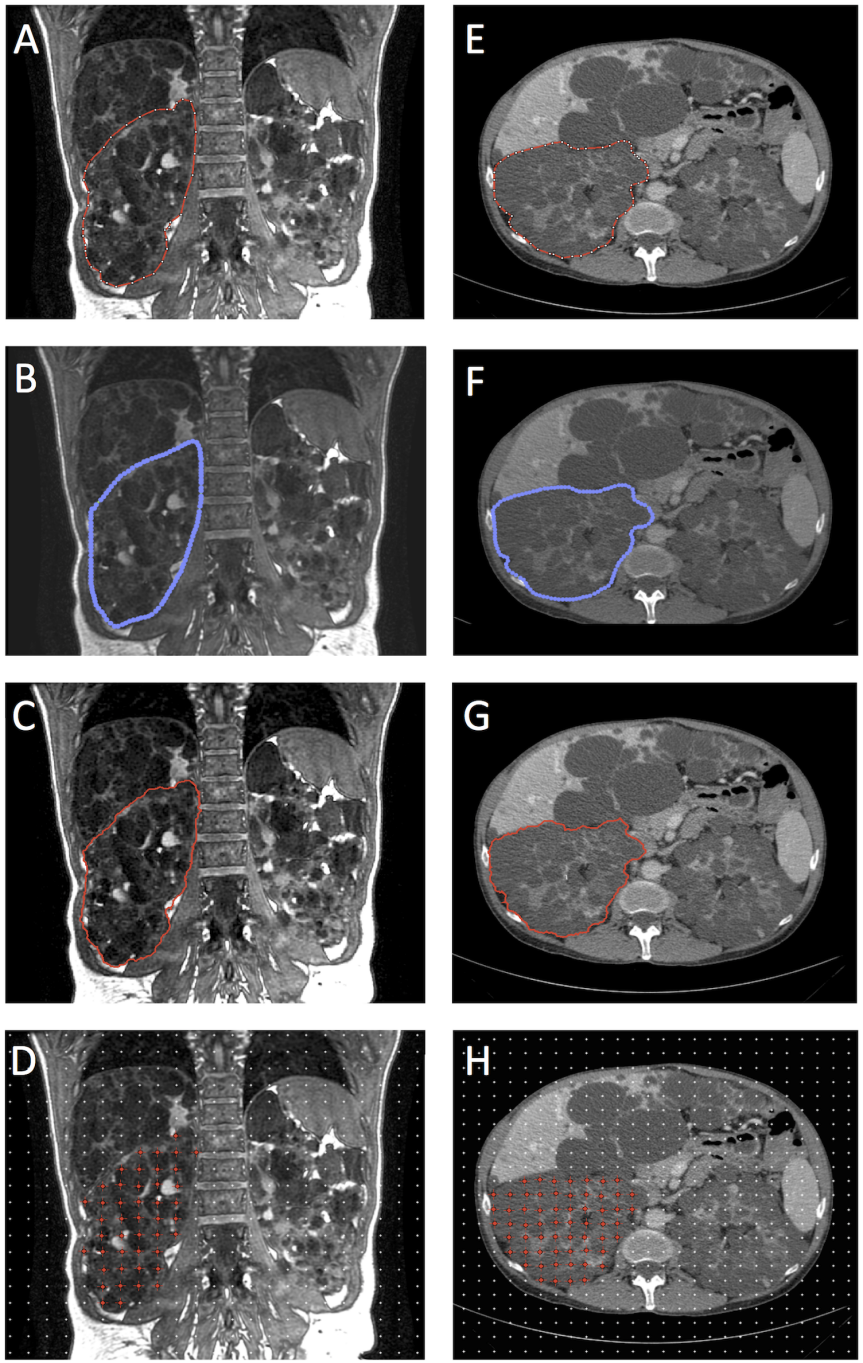
Representative images of polycystic kidney volume segmentations. Segmentation was performed on MRI (panels A-D) and CT image slices (panels E-H) by the expert operator using ImageJ polyline (A and E), Osirix free-hand (B and F), Livewire tool (C and G) and Stereology (D and H).

Representative images of planimetry methods and Stereology, on both MR and CT images, are shown in Fig 1.

#### 2.3.5 Mid-slice method

We then applied the simplified method proposed by Bae and co-workers to estimate SKV using mid-slice images [27]. Briefly, each kidney was outlined on the pertinent mid-slice by manually drawing a polyline, and its volume was estimated by multiplying the mid-slice area by the total number of slices covering the kidney, the slice thickness, and then by an empirically determined factor (0.637 for right kidney, 0.624 for left kidney). This method was originally validated for MR; however, we used the same approach for CT images based on the fact that kidney shape and volume must be similar on MR and CT.

#### 2.3.6 Ellipsoid method

We used the ellipsoid method, proposed by Irazabal and colleagues, to estimate SKV for classification purpose [28]. Briefly, for each kidney separately, length (in both sagittal and coronal orientation), width and depth (in axial orientation) were measured using the Osirix DICOM viewer. SKV was estimated using the following ellipsoid formula. SKV = length (average of sagittal and coronal lengths) x width x depth x (π/6) [28]. An example of SKV assessment by the ellipsoid method is available as Supporting Information (S1 Fig).

### 2.4 Validation study

In the validation study we considered all baseline and 1-year follow-up MR images from the ALADIN study, a long-term clinical study in typical (class 1) ADPKD patients aimed to document the efficacy of Octreotide-LAR (somatostatin analogue) treatment [12]. The patients provided written consent for their medical records to be used in research studies at clinical study enrolment. Data were accessed anonymously. The main socio-demographic and clinical features of ADPKD patients included in the validation dataset (n = 75) are reported in Table 1. Details about estimation of the sample size of the ALADIN study are available in the reference paper [12]. In the ALADIN study, TKV was quantified using ImageJ polyline method. Additionally, in this validation study the expert operator quantified TKV using other available TKV quantification methods (Stereology, Mid-slice and Ellipsoid method), and computed the right and left kidney length. Despite both 1-year and 3-year follow-up MR images being available, 1-year follow-up data were included in the study to investigate the efficacy of TKV quantification methods for detecting small changes developing over short time intervals. We then compared the sensitivity of each TKV quantification methods to detect the difference in TKV change over 1-year period between two treatment groups. Based on the statistics of computed TKV changes, we finally assessed the sample size required by different TKV quantification method to find a significant difference between the two treatment groups.

### 2.5 Statistical analysis

In the experimental dataset, for each SKV quantification method, on MR and CT images separately, reproducibility (intra-rater reliability for both expert and beginner operator) and inter-rater reliability were assessed by coefficients of variation (CVs) for repeated measures [37]. For MR and CT images separately, the agreement between SKV values computed using different methods (within operator, first tracing) was assessed by Bland-Altman plots. The significance of the difference in SKV values and in time required for SKV computation (within operator, first tracing) was assessed by ANOVA (treatment by subjects design), followed by Tukey’s honest significant difference post-hoc test. The same analysis was repeated to assess the significance of the difference in SKV values and time between first and second tracing (within operator). Root mean squared error (RMSE) was used to measure the mean difference between SKV computed by individual computation methods and the reference method (ImageJ polyline). The correlation between SKV and length was assessed using Pearson’s correlation coefficient.

In the validation study, for each KV quantification method, the difference in TKV change between treatment group at 1 year were assessed by ANCOVA, adjusted for baseline measurement. The difference in TKV percentage change was assessed by unpaired t-test. The sample size was computed as minimum size required to assess a significant difference between the two treatment groups based on the mean of the two treatment groups and the standard deviation of the Octreotide-LAR treatment group, assuming type I error = 0.05, and power = 0.80.

Statistical analyses were performed using R software (www.r-project.org), version 3.2.0.

## 3. Results

**Fig 2:**
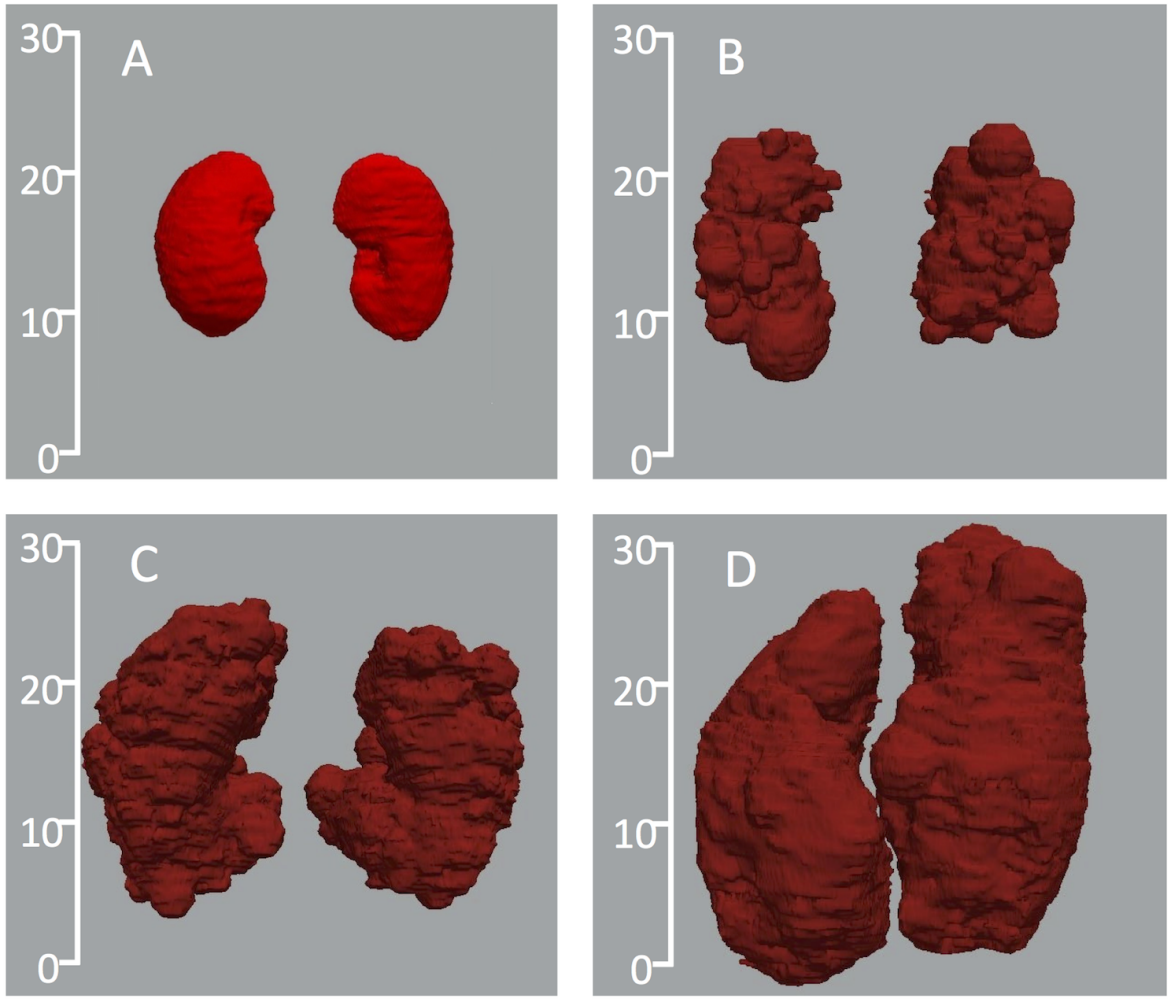
Three-dimensional representation of ADPKD kidneys in comparison with normal kidneys. Scales represent dimension in cm. The kidney shape, size, and volume highly differ between the normal control (panel A: total kidney volume (TKV) = 591 ml) and the patients (panel B: TKV = 1327 ml; panel C: TKV = 3026 ml; panel D: TKV = 5836 ml). Kidneys were reconstructed using VMTK software based on binary masks obtained from ImageJ polyline manual tracings on representative CT images.

As shown by representative images in Fig 2, volume and shape of the ADPKD kidneys can vary considerably. Some kidneys may have regular shape while others are markedly irregular. In some patients, surface irregularities are prominent due to the presence of cysts of different size. This heterogeneity makes KV measurements more difficult and different in each patient.

### 3.1 Intra- and inter-rater agreement

Descriptive statistics of SKV data and processing time required by each method for the two operators during first and second tracing, for the experimental dataset, are reported in Table 2. Intra-operator and inter-operator differences in estimating SKV with all methods are shown in Table 3.

**Table 2:** Single kidney volume (SKV) assessed by different methods and time required by expert and beginner operator on MR and CT images from ADPKD patients in the experimental dataset.

	KV computation method	Expert			Beginner		
		1st tracing	2nd tracing		1st tracing	2nd tracing	
		SKV ml	SKV ml	Time min	SKV ml	SKV ml	Time min
MR	ImageJ polyline	1420±989	1424±988	35±12	1448±998	1460±993	40±14
	Osirix free-hand	1401±975	1410±982	21±9**	1425±974	1419±980	22±08**
	Livewire tool	1410±996	1418±991	26±9*	1445±1009	1438±993	33±15
	Stereology	1373±956	1365±965	15±9**	1407±977	1391±966	19±16**
	Mid-slice method	1401±982	1421±996	9±1**	1462±987	1476±1019	11±1**
	Ellipsoid method	1207±940**	1230±970**	5±1**	1207±1016**	1308±984*	6±0**
CT	ImageJ polyline	1577±921	1572±923	31±11	1584±931	1597±936	52±18
	Osirix free-hand	1579±924	1587±930	18±7**	1572±924	1572±925	28±10**
	Livewire tool	1551±916	1541±910	24±8*	1563±922	1558±913	32±11**
	Stereology	1566±919	1550±908	17±9**	1653±973	1661±968	26±14**
	Mid-slice Method	1387±827**	1368±833**	11±1**	1368±828**	1395±849**	11±1**
	Ellipsoid method	1450±909*	1480±900*	4±1**	1329±849**	1322±819**	5±1**

** p<0.001 and,

* p<0.05 at Tukey’s honest significant difference post-hoc test (individual methods vs reference ImageJ polyline method).

Number of single kidneys analyzed n = 30 for MR and n = 30 for CT. Single Kidney volumes (SKV) are expressed as mean ± SD.

SKV (ml) were computed by both operators (expert and beginner), two weeks apart (1^st^ tracing vs. 2^nd^ tracing).

Time (min) was estimated on total kidney volumes (sum of right and left SKV).

**Table 3:** Inter and intra-rater reproducibility of single kidney volume (SKV) measured by expert and beginner operators using different quantification methods on MR and CT images from ADPKD patients in the experimental dataset.

	SKV computation method	Intra-rater (Expert)			Intra-rater (Beginner)			Inter-rater		
		SKV Difference ml	SKV Difference %	CV %	SKV Difference ml	SKV Difference %	CV %	SKV Difference ml	SKV Difference %	CV %
MR	ImageJ polyline	-4±24	-0.4±2.4	1.18	-12±84	-3.3±18.3	4.04	-28±50	-2.5±4.7	2.77
	Osirix free-hand	-9±24	-0.3±2.5	1.26	7±116	0.8±7.4	5.70	-24±97	-3.1±7.1	4.92
	Livewire tool	-8±24	-1.3±3.4	1.25	7±47	-0.5±3.7	2.28	-35±46	-2.8±4.6	2.83
	Stereology	7±55	1.3±5.5	2.80	16±82	0.2±11	4.13	-34±89	-2.9±14.6	4.75
	Mid-slice method	-20±60	-2.2±7.7	3.11	-13±115	-0.8±5.1	5.48	-62±252	-11.3±29.8	12.61
	Ellipsoid method	-23±146	-3.8±25.3	8.46	-101±367	-19.1±46	21.06	-0.79±242	0.1±31.9	17.73
CT	ImageJ polyline	6±25	0.6±1.3	1.14	-13±31	-1.2±1.9	1.48	-7±31	-0.03±2.0	1.38
	Osirix free-hand	-8±19	-0.5±1.3	0.89	-0.1±27	-0.3±2.0	1.19	7±20	0.7±1.6	0.95
	Livewire tool	10±21	0.7±1.1	1.05	5±19	-0.2±1.9	0.88	-12±25	-0.8±1.6	1.23
	Stereology	15±35	0.4±2.9	1.69	-9±24	-1.2±3.0	1.07	-87±66	-5.6±3.9	4.77
	Mid-slice method	19±117	1.6±6.3	6.00	-26±58	-2.0±3.6	3.21	18±73	1.8±4.6	3.79
	Ellipsoid method	-31±193	-4.1±16.9	9.28	7±185	0.6±12.3	9.70	121±287	5.9±18.0	15.65

Number of single kidneys analyzed n = 30 for MR and n = 30 for CT. Single Kidney Volume (SKV) difference is expressed as mean ± SD.

SKV Difference (ml) = Absolute difference between 1^st^ and 2^nd^ tracing; SKV Difference (%) = Percentage difference between 1^st^ and 2^nd^ tracing; CV = coefficient of variation for repeated measures.

Planimetry methods and Stereology showed the highest intra- and inter-rater agreement for both MR and CT images. For MR, the CVs were consistently lower for the expert operator compared to the beginner. For CT, the two operators had similar CVs, suggesting that reliable identification of kidney contour by non-expert operator is easier on CT than on MR.

For the expert operator, all three planimetry methods were more reproducible than Stereology and Mid-slice method, on MR as well as CT, while the Ellipsoid method had the lowest reproducibility. For the beginner operator, the Livewire tool was the most reproducible while the Ellipsoid method was the least reproducible method. No significant difference in SKV values and time was found between first and second tracing (within operator).

The inter-rater performance on MR was generally worse than CT (Table 3). On MR, the ImageJ polyline and Livewire tool had the lowest CV, followed by Stereology and Osirix free-hand. The inter-rater performance on CT was less variable and the method with the lowest CV was Osirix free-hand, followed by the Livewire tool, ImageJ polyline, Mid-slice method, and Stereology. The Ellipsoid method had the lowest inter-rater reproducibility on both MR and CT (Table 3). No consistent difference in reproducibility was found between left and right kidneys.

### 3.2 Comparison of different methods

**Fig 3:**
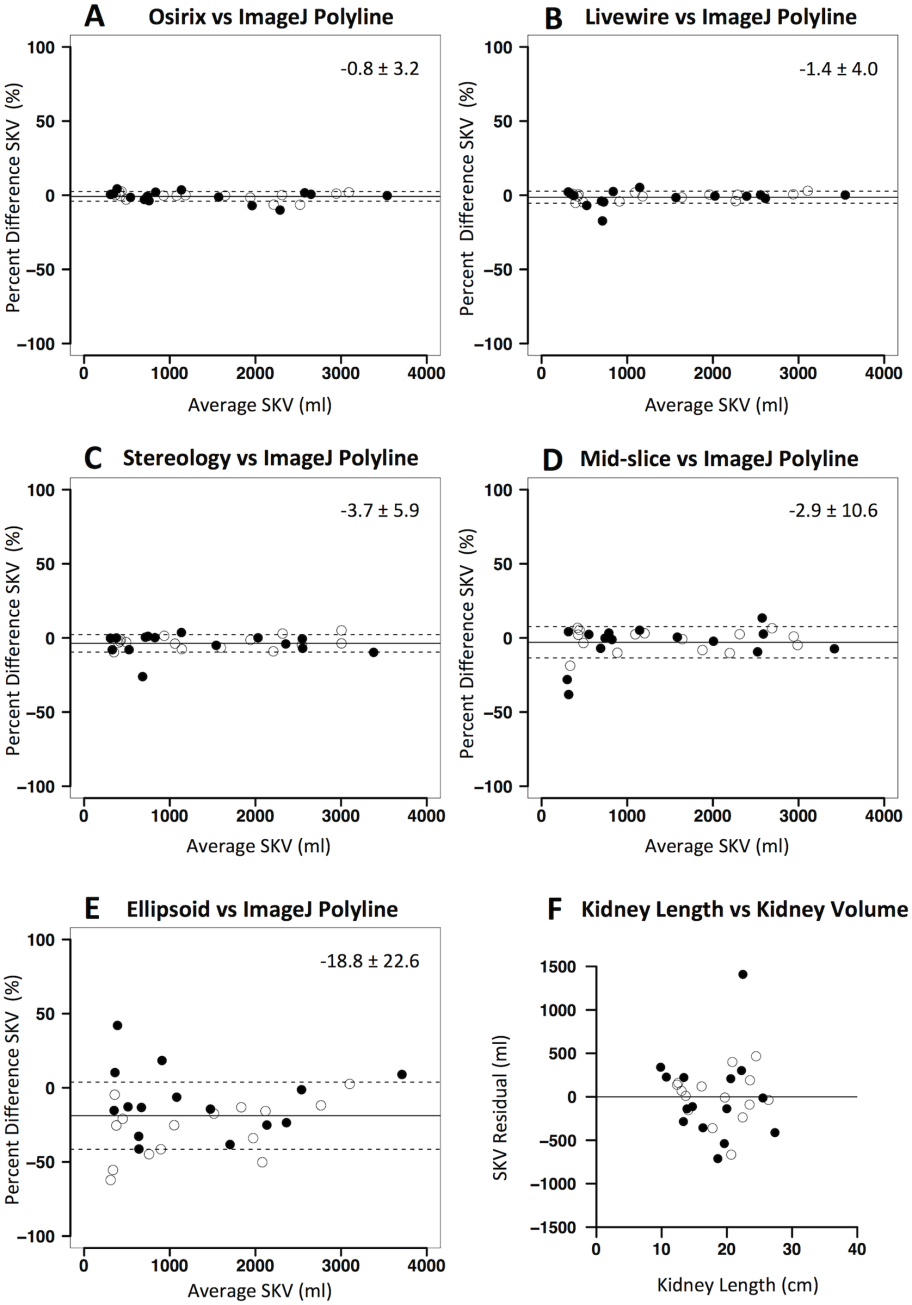
Agreement between kidney volume computation methods on MR in the experimental dataset. Panels A-E: Bland-Altman plots showing agreement between different kidney volume computation methods (A: Osirix free-hand; B: Livewire tool; C: Stereology; D: Mid-slice method; E: Ellipsoid method) versus ImageJ polyline (reference method). Percent differences in single kidney volume (SKV) are plotted against average SKV values of the two methods. Solid lines denote mean difference, while dashed lines denote ± standard deviations. Panel F: plot of the residual of the linear regression of kidney length against SKV (assessed by reference ImageJ polyline method). Black dots represent right kidneys while white dots represent left kidneys.

On MR images, Osirix free-hand showed the highest agreement with ImageJ polyline (adopted as the reference method as mentioned previously) (Table 4). The accuracy of the method was high (mean difference of -0.8%) and estimated precision (percentage root mean square error, RMSE) was equal to 3.2%. The Livewire tool showed high accuracy and precision, despite being lower than Osirix free-hand. Stereology showed even lower accuracy (mean difference of -3.7%) and inferior precision (percentage RMSE equal to 6.3%). The agreement between different methods is shown in Bland-Altman plots reported in Fig 3. Simplified methods showed the lowest accuracy and precision (as shown in Table 4 and in Fig 3) and the difference in SKV between Ellipsoid method and ImageJ polyline (mean of -18.8%) was statistically significant (p< 0.01).

**Table 4:** Absolute and percentage difference and root mean squared error (RMSE) between methods used to compute single kidney volume (SKV) by the expert operator on MR and CT images from ADPKD patients in the experimental dataset.

	SKV computation method	SKV Difference ml	RMSE ml	SKV Difference %	RMSE %
MR	Osirix free-hand vs ImageJ polyline	-19 [–229, 60]	66	-0.8 [-9.5, 4.4]	3.2
	Livewire tool vs ImageJ polyline	-10 [–123, 93]	40	-1.4 [-15.9, 5.4]	4.0
	Stereology vs ImageJ polyline	-47 [–329, 152]	101	-3.7 [-23.1, 5.2]	6.3
	Mid-slice vs ImageJ polyline	-19 [–251, 346]	118	-2.9 [-32.1, 14.4]	9.7
	Ellipsoid vs ImageJ polyline	-213 [–1044, 333]	350	-18.8 [-48.0, 53.2]	25.4
CT	Osirix free-hand vs ImageJ polyline	2 [–40, 82]	23	-0.1 [-2.7, 3.2]	1.3
	Livewire tool vs ImageJ polyline	-26 [–88, 42]	39	-2.2 [-6.2, 1.6]	2.8
	Stereology vs ImageJ polyline	-11 [–67, 39]	26	-1.0 [-5.2, 3.2]	2.1
	Mid-slice vs ImageJ polyline	-190 [–862, 23]	276	-13.4 [-27.1, 2.8]	15.0
	Ellipsoid vs ImageJ polyline	-128 [–666, 559]	289	-10.7 [-33.6, 34.5]	19.0

Number of single kidneys analyzed n = 30 for MR and n = 30 for CT. SKV are from first tracing of expert operator.

SKV difference is expressed as mean difference and [range]; RMSE = Root mean square error.

**Fig 4:**
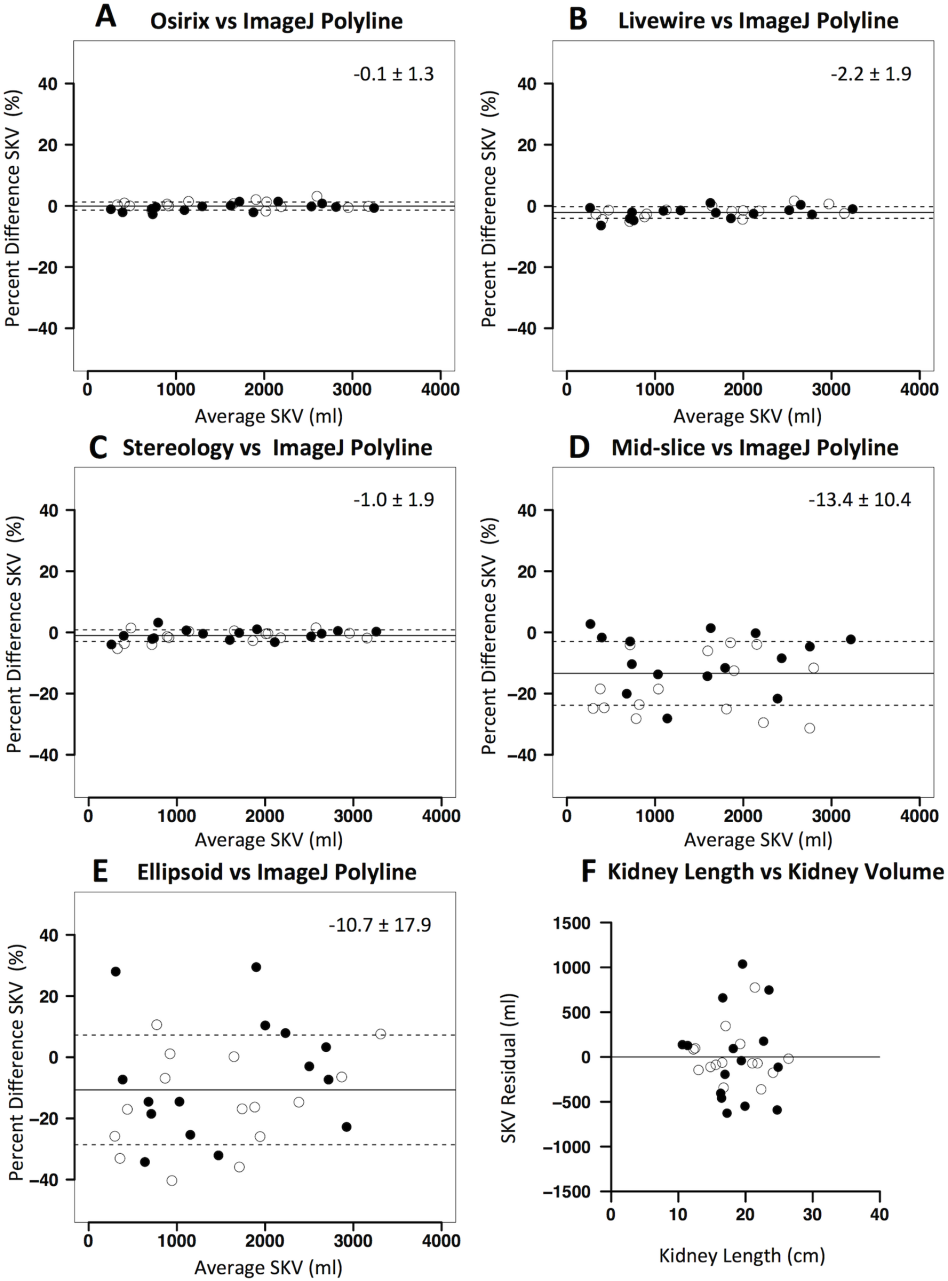
Agreement between kidney volume computation methods on CT in the experimental dataset. Panels A-E: Bland-Altman plots showing agreement different kidney volume computation methods (A: Osirix free-hand; B: Livewire tool; C: Stereology; D: Mid-slice method; E: Ellipsoid method) versus ImageJ polyline (reference method). Percent differences in single kidney volume (SKV) are plotted against average SKV values of the two methods. Solid lines denote mean difference, while dashed lines denote ± standard deviations. Panel F: plot of the residual of the linear regression of kidney length against SKV (assessed by reference ImageJ polyline method). Black dots represent right kidneys while white dots represent left kidneys.

The results of the comparison between different methods and ImageJ polyline method on CT was similar to that reported for MR. Thus, Osirix free-hand method and the Livewire tool were the best performing methods (Table 4 and Fig 4) with respect to the accuracy and precision. Stereology was as precise and accurate as the planimetry methods (Table 4). The simplified methods were neither accurate nor precise (Table 4 and Fig 4). The difference between SKV measured with these methods and ImageJ polyline was statistically significant (p<0.01 for Mid-slice method; p<0.01 for Ellipsoid method).

At single kidney level, organ length showed an expected positive and significant correlation with SKV (ImageJ polyline, r = 0.91; p<0.01 and r = 0.90; p<0.01 on MR and CT, respectively). However, the residual plot of the correlation analysis demonstrates that kidney length is far from being precise as required, for both imaging modalities (see Figs 3 and 4). Again, no consistent difference in agreement between methods was found between left and right kidneys.

### 3.3 Time requirement

For both MR and CT images, and for both expert and beginner operators, ImageJ polyline method required the longest time (Table 2) with more than 30 min on average. The Livewire tool required relatively shorter time on both MR and CT, but the reduction was only 8 min on average. Osirix free-hand was the fastest among planimetry methods for both operators, reducing the mean time to only 20 and 16 min on MR and CT, respectively. As expected, shorter time was required for Stereology, with an average of 11 and 14 min for MR and CT, respectively. Moreover, the time required for KV measurement was consistently higher for the beginner operator (Table 2). Finally, the simplified methods (Mid-slice and Ellipsoid methods) were very fast and required the shortest time (approximately 10 and 5 min, respectively).

### 3.4 Validation study

The results of the validation study are shown in Table 5. As previously reported [12], when TKV was quantified by ImageJ polyline, both absolute and percentage changes in TKV at 1 year of treatment were significantly different between ADPKD patients under Octreotide-LAR (n = 38) and placebo treatment (n = 37) (p = 0.032 and p = 0.003, respectively). The between-treatment difference in TKV change remained statistically significant when TKV was quantified by Stereology (absolute and percentage change: p = 0.018 and p = 0.016, respectively), while it was not statistically significant when TKV was estimated by Mid-slice or Ellipsoid methods (Table 5). Similarly, the between-treatment difference in kidney length (computed as sum of right and left kidney lengths) was not statistically significant (Table 5), indicating that simplified methods did not identify between-treatment changes occurring over the treatment period.

**Table 5:** Total kidney volume changes compared with baseline at 1 year of treatment with placebo or Octreotide-LAR. Total kidney volume was assessed by different kidney volume computation methods on MR images taken from the ALADIN clinical study [12].

	Absolute change in TKV (ml)			Percentage change in TKV (%)		
KV computation method	Octreotide-LAR (n = 38)	Placebo (n = 37)	p	Octreotide-LAR (n = 38)	Placebo (n = 37)	p
ImageJ polyline	46.1 ±112.3	143.7 ±158.1	0.032 (<0.05)	2.57 ±6.07	6.72 ±5.89	0.003 (<0.01)
Stereology	45.8 ±114.1	152.1 ±160.4	0.018 (<0.05)	3.30 ±7.14	7.00 ±5.83	0.016 (<0.05)
Mid-slice method	40.1 ±129.8	127.2 ±186.0	0.111 (NS)	2.89 ±10.71	6.55 ±8.06	0.098 (NS)
Ellipsoid method	36.3 ±153.9	125.4 ±179.1	0.102 (NS)	2.54 ±11.66	6.35 ±9.99	0.132 (NS)
Kidney length^#^	-0.25 ±1.22	0.26 ±1.97	0.115 (NS)	-0.69±3.97	1.10 ±6.69	0.165 (NS)

Abbreviations: LAR, long-acting release; KV, kidney volume; NS, not statistically significant; TKV, total kidney volume (sum of right and left kidney volumes).

^#^ Kidney length (in cm) is computed as sum of right and left kidney lengths.

p values from ANCOVA (absolute change) or unpaired t-test (percentage change).

Based on the statistics of computed TKV percentage changes, we calculated that the sample size required by different TKV quantification methods to find a significant difference between the two treatment groups is lowest for ImageJ polyline (n = 34 per treatment group), followed by Stereology (n = 59). The estimated sample size greatly increases, up to 4-fold, in case TKV is measured by Mid-slice (n = 135) or Ellipsoid method (n = 147).

## 4. Discussion

In this study we quantitatively compared different methods for KV computation in terms of reproducibility, accuracy, precision and time required on both MR and CT representative images. The experimental set included a series of 30 kidneys for each imaging modality, with a wide range of SKV values. Overall, planimetry methods and Stereology showed high reproducibility, low bias, and excellent accuracy and precision.

Planimetry methods showed the highest reproducibility. However, high intra-rater variability for the beginner operator suggests that KV computation on MR needs to be performed by expert operators, to reliably detect KV changes. The reproducibility of planimetry and Stereology was worst for MR than CT, and this is likely due to lower image quality on MR compared to CT, making kidney identification on MR more operator-dependent. The Mid-slice and Ellipsoid methods, despite providing quick KV estimates, are less reproducible.

The accuracy and precision of the KV computation methods assessed, adopting ImageJ polyline as reference method, was different for MR and CT images. On MR, planimetry showed highest accuracy and precision, while on CT Stereology performed equally well. Beyond higher image quality, more number of axial sections likely leads to higher precision and accuracy of Stereology on CT. The Mid-slice and Ellipsoid methods showed lowest precision and accuracy on both MR and CT images. This is in line with a previous report that compared the Ellipsoid method with Stereology on MR or CT images covering a large KV range [28]. The study showed important discrepancies between the two methods, with a CV of more than 10%. In addition, we found that the Ellipsoid method consistently underestimates KV, in agreement with another recent study comparing the Ellipsoid method with planimetry on MR [32]. The Mid-slice method was previously compared to Stereology on MR images in terms of correlation, with important differences in absolute values between the two methods [27]. A recent study compared both Ellipsoid and Mid-slice methods with planimetry [29]. The Authors of this study found important differences in accuracy (expressed as bias by these Authors) and precision between the methods (0.2% and 3.2% for repeated manual tracing; 1.4% and 9.2% for Ellipsoid method; 4.6% and 7.6% for Mid-slice method, respectively). Moreover, Ellipsoid method showed lower agreement with planimetry than Mid-slice method [29], in line with our findings on MR. Thus, our present data and those reported by other investigators indicate that both Mid-slice and Ellipsoid methods can hardly detect changes of 3 to 5% in KV since their precision (i.e. SD of the difference between KV calculated by these methods and the reference method) ranges between 10 and 25%. In addition, the results of our validation study indicate that Mid-slice and Ellipsoid methods are not as precise as required for clinical studies to identify between-treatment changes in TKV that occurred over a one-year treatment period in our previous clinical investigation. Indeed, we found that these methods, due to the high variability in estimating TKV, would require a 4-fold larger sample size than ImageJ polyline to find a significant difference between TKV changes in the two treatment groups. Of interest, also Stereology showed a high performance, allowing detection of statistically significant difference in TKV between the treated and control groups.

Alternatively to SKV measurement, kidney length has recently been proposed as predictor of disease progression [31], as this parameter can easily be obtained by ultrasounds investigation. As expected, kidney length and volume, assessed on either MR or CT, are linearly correlated. However, this correlation is characterized by very low precision. Thus, kidney length may only be used to roughly estimate TKV. In addition, our validation study shows that kidney length is not accurate enough to identify between-treatment changes, suggesting that it should not be recommended as outcome measure for clinical trials.

Despite being precise and accurate, planimetry requires 20 to 40 min on average (21 to 35 min for expert operators, for two kidneys). The fastest planimetry method was the Osirix free-hand (only 21 min on average on MR and 18 min on CT). The Stereology technique allows reducing average time to 15 min for MR, and 17 min for CT. The time required is further reduced by simplified methods (5 to 10 minutes approximately), but as mentioned before with the detriment to precision and accuracy of the measurement.

To overcome time requirement and operator-dependency limiting the planimetry methods, it would be ideal to use completely automated methods. However, despite several attempts reported in literature, to develop automatic segmentation tools that attain required accuracy and precision is challenging. Some investigators recently reported new methods for automatic measurements of KV [38–44]. The performance of these methods is highly dependent on image quality and thus automatic tools may fail rather frequently. Moreover, automatic segmentations show high variability and rather large differences with manual contouring [38]. Thus, so far, automatic methods are not accurate enough and, therefore, cannot replace planimetry to detect small KV changes in time, as those expected in clinical trials.

Our study has to be interpreted in the context of the methods we adopted. The main limitation is the restricted number of kidneys from ADPKD patients in the experimental dataset. However, our objective was to estimate the variability of the difference of KV measurements within/between operators and among different computation methods. It has been shown that using a sample size of 30 patients, the SD of a population of measures is estimated with a power of 80% in a range of ±17% [45]. We think that this level of accuracy was enough to appreciate variability of the methods and to support our conclusion. Second, in this study we included both non-contrast enhanced MR and iodinated contrast enhanced CT imaging data. However, it should be noted that performing contrast enhanced CT imaging for clinical studies is a difficult issue, because of the possibility of contrast nephropathy and radiation burden. MR and CT images used in the study, already available from clinical studies, were from different ADPKD patients. In case both MR and CT from the same subjects were available, it would be interesting to directly compare SKV assessed on different image modalities by different methods. Moreover, CT images were from ADPKD patients at a more advanced stage of the disease, who are likely to have kidneys more distorted in shape than patients at an earlier stage. This difference could have affected the comparison between imaging modalities in terms of method reproducibility and accuracy. In addition, as KV range is very broad, there could be a difference in reproducibility or accuracy between small and larger kidneys. It is possible that smaller kidneys had a higher inter- and intra-rater agreement than larger kidneys, and more simplified techniques could possibly be used in this patient group with higher accuracy. Last, despite liver volume being one of the endpoints of clinical studies on ADPKD, a systematic comparison of the different methods available for liver volume computation was beyond the scope of the study.

In conclusion, the results of our study indicate that planimetry methods are more reproducible, accurate and precise than other simpler methods (i.e. Mid-slice and Ellipsoid method) and, despite being more time consuming, they should be preferred for accurately monitoring ADPKD progression and assessing the effects of drug treatments, especially on MR, since they require an importantly lower number of patients to be enrolled in clinical investigations. A good alternative to planimetry methods is Stereology. The fastest simplified methods can provide useful information for ADPKD patient classification but they are less efficient for computation of TKV as end-point of clinical investigations. Our data, even if based on only one expert and one beginner operator, also suggest that in clinical studies multi-centric quantification of TKV should be performed using a single method and should be performed by expert operators, especially when processing MR images.

## Supporting information

S1 Fig Example single kidney volume (SKV) assessment using the Ellipsoid method. SKV assessment was performed by the expert tracer on MR (panel A, left to right: coronal, sagittal, and axial view) and CT (panel B, left to right: coronal, sagittal, and axial view). Kidney length was assessed on both coronal and sagittal planes, while kidney depth and width were assessed on axial plane. Kidney volume was estimated using the following ellipsoid formula: kidney volume = length (average of sagittal and coronal lengths) x width x depth x (π/6).
(TIF)

S1 File Appendix I. Estimation of ellipsoid volume by planimetry.
(PDF)

S2 File Experimental dataset. (XLSX)

S3 File Validation dataset. (XLSX)

## Abbreviations

ADPKD: Autosomal dominant polycystic kidney disease
CT: computed tomography
CV: coefficient of variation
GFR: glomerular filtration rate
MR: magnetic resonance
RMSE: root mean squared error
SKV: single kidney volume
TKV: total kidney volume
