# Supplementary figures and images for: Kidney volume measurement methods for clinical studies on autosomal dominant polycystic kidney disease
Source: PLoS One. 2017 May 30;12(5):e0178488. doi: 10.1371/journal.pone.0178488 (PMC5448775; doi:10.1371/journal.pone.0178488)

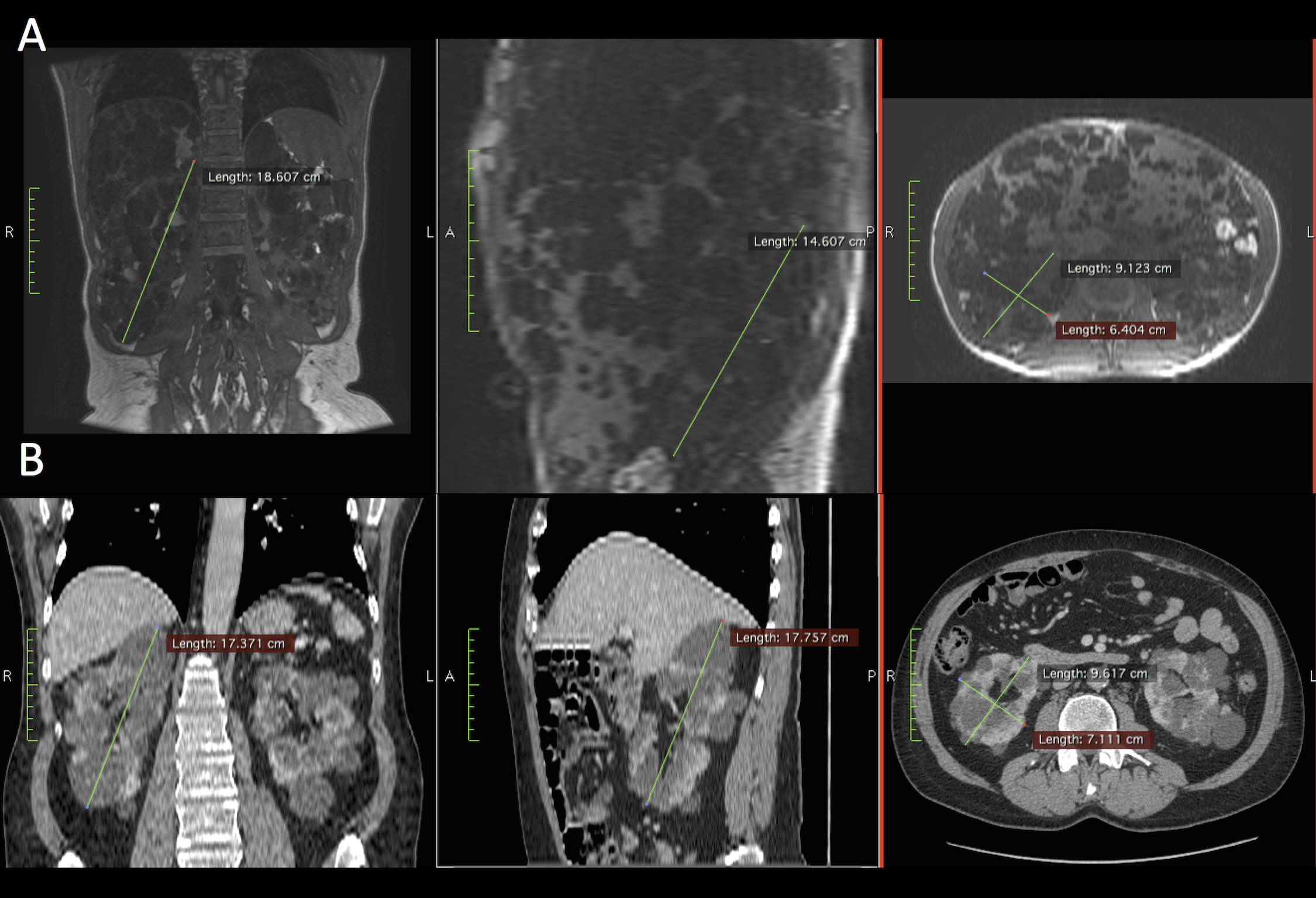

Supplement: S1 Fig — SKV assessment was performed by the expert tracer on MR (panel A, left to right: coronal, sagittal, and axial view) and CT (panel B, left to right: coronal, sagittal, and axial view). Kidney length was assessed on both coronal and sagittal planes, while kidney depth and width were assessed on axial plane. Kidney volume was estimated using the following ellipsoid formula: kidney volume = length (average of sagittal and coronal lengths) x width x depth x (π/6). (TIF) [file pone.0178488.s001.tif]
