# Supplementary material for: Kidney volume measurement methods for clinical studies on autosomal dominant polycystic kidney disease
Source: PLoS One. 2017 May 30;12(5):e0178488. doi: 10.1371/journal.pone.0178488 (PMC5448775; doi:10.1371/journal.pone.0178488)
Supplement: S1 File — Estimation of ellipsoid volume by planimetry. (PDF) [file pone.0178488.s002.pdf]

Appendix I

Estimation of ellipsoid volume by planimetry

The estimation of the volume of an ellipsoid, the idealized volume of a kidney, by planimetry is based on the calculation of the area of equispaced sections perpendicular to one axis. As shown in Fig A1, while the volume of an ellipsoid can be estimated by three semi-axes (a, b, c) by the equation

$$V = \frac{4}{3}\pi\, a\, b\, c$$

using planimery the volume (V_p) can be estimated by the equation

$$V_p = \sum_{1}^{N} A_i\, d$$

where A_i is the area of section i and N is the number of equally spaced sections of thickness d. The estimation of the ellipsoid volume can depend on section thickness and on section orientation. Since estimation of kidney volume by planimetry is related to slice thickness, as well as to orientation of kidney sections, we estimated the planimetry error based on ellipsoids of volumes comparable to ADPKD kidney volumes, using slice thickness and orientation corresponding to MR and CT images.

In detail, we divided each of the two groups of ADPKD patients studied with MR and CT, respectively, in 3 subgroups based on kidney volumes estimated by polyline manual tracing method (as reported in Table A1). We then computed the mean major axis (length) of the three kidney volume classes, and the maximum area perpendicular to the major axis (Table A1).

Table A1: **Single kidney volume (SKV), maximum area and length of average kidneys of different size.**

		**SKV** (ml)	**Max area** (mm^2)	**Length** (mm)
**MR**	**Small** (n=10)	370	4,049	135.6
	**Medium** (n=10)	973	7,364	192.4
	**Large** (n=10)	2277	13,541	247.2
**CT**	**Small** (n=10)	503	5,192	150.5
	**Medium** (n=10)	1297	9,973	204.0
	**Large** (n=10)	2354	14,752	252.5

We then considered 6 ellipsoids representative of the three average kidney volumes derived from MR and CT images, assuming length and semi-axes reported in Table A2. Ellipsoid volumes computed using analytical equation are reported in Table A2.

Table A2: **Geometrical parameters of ellipsoids assumed to be representative of ADPKD kidneys of different size**

	**Max area** (mm^2)	**a** (mm)	**b, c** (mm)	**Volume** (ml)
**MR**	4,071	67.5	36	366
	7,854	95	50	994
	13,685	125	66	2,280
**CT**	5,024	75	40	503
	9,498	100	55	1266
	14,306	125	67.5	2,384

To simulate the effect of planimetry tracing on the estimation of ellipsoid volume, we computed the area of ellipsoid sections, with transversal or longitudinal orientation (representative of MR and CT image sequences) and section thickness of 4 and 5 mm for MRI and CT, respectively (as shown in Fig A1).

Fig A1: **Ellipsoid volume assessment using planimetry.** The ellipsoid is divided in slices (axial and sagittal slices for CT and MR, respectively), and the volume is computed as sum of the slice areas multiplied by the slice thickness.

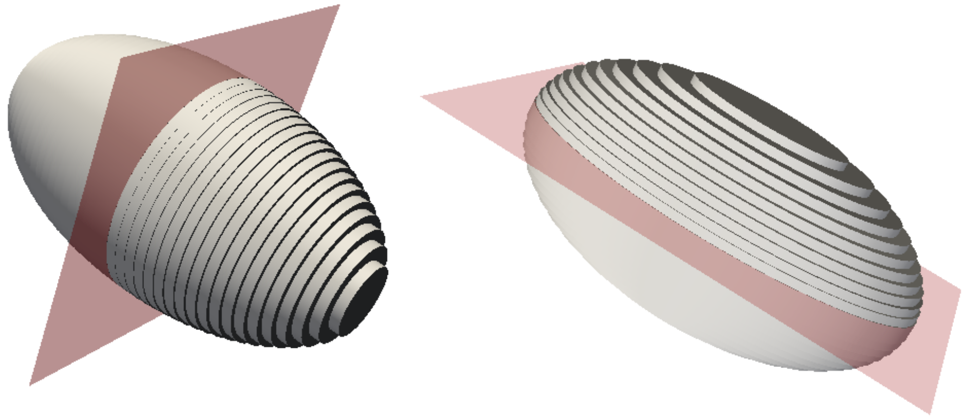

To calculate the radius of the circumferences of the hypothetical ellipsoid sections, for each slice we computed the *y* coordinate of the ellipse equation for a given *x* coordinate as

$$y_T = \sqrt{(1 - \frac{x^2}{a^2})b^2} \qquad \text{and} \qquad y_L = \sqrt{(1 - \frac{x^2}{b^2})a^2}$$

for transverse and longitudinal sections, respectively. We then calculated the slice area $A_i = \pi y_i^2$. We then computed the ellipsoid volume as sum of the areas of all ellipsoid sections multiplied by the slice thickness. Ellipsoid volumes computed by planimetry are reported in Table A3. Finally we calculated the error between analytical ellipsoid volume and the volume estimated by planimetry (see Table A3). We repeated the calculation using different slicing offsets, in order to quantify the error due to random slice positioning.

Table A3: **Example ellipsoid volumes computed by planimetry, and percentage errors with respect to analytical volumes.** Since errors slightly change with the slicing offset, minimum and maximum errors are reported.

		**Volume by planimetry** (ml)	**Volume difference analytical vs. planimetry** (ml)	**Error** (%) Min / Max
**MR**	**Small**	367.0	-0.75	-0.20 / +0.26
	**Medium**	993.2	1.08	-0.13 / +0.11
	**Large**	2,278.7	0.94	-0.10 / +0.04
**CT**	**Small**	502.1	0.30	-0.10 / +0.06
	**Medium**	1,266.3	0.15	-0.08 / +0.01
	**Large**	2,384.7	-0.25	-0.07 / -0.01

The difference between analytical and planimetry volume is very small, with a percentage error less than 0.3% (Table A3), suggesting that the estimation of the volume of ellipsoids representative of ADPKD kidneys of different sizes can be reliably obtained by planimetry, both for orientation and section thickness of MR and CT image sequences.
